# Overuse of tympanostomy tubes in New York metropolitan area: evidence from five hospital cohort

**DOI:** 10.1136/bmj.a1607

**Published:** 2008-10-03

**Authors:** Salomeh Keyhani, Lawrence C Kleinman, Michael Rothschild, Joseph M Bernstein, Rebecca Anderson, Mark Chassin

**Affiliations:** 1 Department of Health Policy, Mount Sinai School of Medicine, One Gustave L Levy Place, Box 1077, New York, NY 10029, USA; 2 Departments of Otolaryngology and Pediatrics, Mount Sinai School of Medicine; 3 Departments of Otolaryngology and Pediatrics, New York University School of Medicine, New York; 4 The Joint Commission, Oakbrook Terrace, IL, USA

## Abstract

**Objectives** To compare tympanostomy tube insertion for children with otitis media in 2002 with the recommendations of two sets of expert guidelines.

**Design** Retrospective cohort study.

**Setting** New York metropolitan area practices associated with five diverse hospitals.

**Participants** 682 of 1046 children who received tympanostomy tubes in the five hospitals for whom charts from the hospital, primary care physician, and otolaryngologist could be accessed.

**Results** The mean age was 3.8 years. On average, children with acute otitis media had fewer than four infections in the year before surgery. Children with otitis media with effusion had less than 30 consecutive days of effusion at the time of surgery. Concordance with recommendations was very low: 30.3% (n=207) of all tympanostomies were concordant with the explicit criteria developed for this study and 7.5% (n=13) with the 1994 guideline from the American Academy of Pediatrics, American Academy of Family Medicine, and American Academy of Otolaryngology—Head and Neck Surgery. Children who had previously had tympanostomy tube surgery, who were having a concomitant procedure, or who had “at risk conditions” were more likely to be discordant.

**Conclusions** A significant majority of tympanostomy tube insertions in the largest and most populous metropolitan area in the United States were inappropriate according to the explicit criteria and not recommended according to both guidelines. Regardless of whether current practice represents a substantial overuse of surgery or the guidelines are overly restrictive, the persistent discrepancy between guidelines and practice cannot be good for children or for people interested in improving their health care.

## Introduction

Otitis media is the most common illness for which children present to the doctor, and tympanostomy tubes are the most common reason for general anaesthesia in children.^1^ ^2^ ^3^ Otitis media may be characterised by acute otitis media, otitis media with effusion, or both. Otitis media is often recurrent and is consequential in terms of healthcare use.^4^ ^5^ ^6^ ^7^ ^8^ ^9^ ^10^ ^11^ ^12^ ^13^ In a cohort of 2253 children in Pittsburgh, Pennsylvania, 6% of all children had received tympanostomy tubes before their second birthday.^14^ In the United Kingdom, Black described an epidemic in surgery for the treatment of otitis media and extensive geographical variation in the use of the procedure in the 1980s.^15^ ^16^ Black and Hutchings also reported that the dissemination of guidelines in the 1990s may have accelerated a trend towards decreased use of surgery in the UK, although a trend towards decreased use was already apparent even before the publication of a guideline.^17^ Extensive variation in the regional use of this procedure has also been described in Canada, Finland, and Norway.^18^ ^19^ ^20^ No recent studies have looked at the use of this surgery in the United States, but in 1996 more than half a million tympanostomies were done.^21^

Although many studies have assessed the degree to which the use of procedures in adults is concordant with guidelines,^22^ ^23^ ^24^ studies in children are less common. Only one study has examined the appropriateness of insertion of tympanostomy tubes in practice.^4^ ^5^ This study, published in 1994, reported that less than half of surgeries among children in the United States were appropriate. Since then, several guidelines on the management of otitis media have been published.^2^ ^25^ ^26^ In this paper, we compare the clinical characteristics of the children in our study with the recommended indications for surgery as codified by the prevailing guideline at that time (the 1994 guideline on otitis media with effusion developed by the American Academy of Pediatrics, American Academy of Family Medicine, and American Academy of Otolaryngology—Head and Neck Surgery^2^ ^26^) and a set of explicit criteria that we developed in 2000 specifically for this study as an update to the 1994 guidelines by using the RAND appropriateness method. To our knowledge, this is the first study to examine the appropriateness of insertion of tympanostomy tubes with data collected by independent audits of the records.

## Methods

### Study population and data

We did a retrospective cohort study and collected detailed data for a one year period before insertion of a tympanotomy tube for each child. We have previously described our population and data collection methods in detail.^27^ We developed an electronic tool to assist data collection for chart audits of all 1046 children less than 18 years old who were identified by hospital administrative databases as having received tympanostomy in any of five New York metropolitan area hospitals in 2002. Data collection began in 2003 and was completed in 2005. This paper reports on those 682 children for whom we were able to audit all three medical records: from the primary care physician’s office, the otolaryngologist’s office, and the hospital. We have previously shown that the children who were excluded owing to incomplete data did not differ in sociodemographic characteristics from those who were included.^27^ The five hospitals included two academic medical centres, one tertiary care teaching hospital, one private not for profit community hospital, and one public teaching hospital.^27^

Clinical data abstracted from the chart included dates of service, otoscopic findings, previous history of otitis media, treatment with antibiotics, clinicians’ diagnosis of acute otitis media or otitis media with effusion, documented hearing loss, notation about the impact of otitis media on family life, and the presence of conditions that may be considered to put the child “at risk” for worse outcomes (autism, developmental delay, Down’s syndrome, craniofacial syndromes that include cognitive, speech or language delay, or visual impairment).^27^ When hearing loss was present, we dichotomised it as mild (20-35 dB loss in the best ear) or moderate to severe (>35 dB loss in the best ear).^27^ For the rare (1%) children for whom the physician documented hearing loss but a formal assessment was absent from all records, we considered hearing loss to be present. Similarly, we considered the documentation of a parent’s or physician’s assertion of speech or language delay without a formal assessment to be sufficient evidence, unless a subsequent assessment documented normal speech before surgery.^27^ We considered severe disruptions of family life to be present on the basis of documentation indicating missed school or work, a comment about an excessive number of physicians’ appointments, serious disturbances in the family’s usual affairs, or considerable anxiety about the impact of ear disease any time in the three months leading up to surgery.^27^ We made two explicit assumptions to guide our interpretation of the data from medical records: unless otherwise documented, we postulated that otitis media with effusion persisted for 60 days after any documentation and that otoscopic findings did not return to normal for 28 days after acute otitis media. For example, we considered a child who had otitis media with effusion documented on day 1 and on day 50 to have had an effusion for 110 consecutive days (50 plus 60) if no other examinations were documented. We identified surgeries or procedures that were done concurrently with insertion of tympanostomy tubes from hospital administrative data.

### Development of explicit criteria

The RAND appropriateness method uses a two round modified Delphi process to integrate literature with expert opinion into explicit criteria,^28^ in this case rating the appropriateness of tympanostomy tubes for children under 18 years old. We convened an expert panel of four otolaryngologists, four paediatricians, and one family physician and provided them with a detailed literature review. The panel identified relevant clinical factors that we organised into an exhaustive and mutually exclusive list of potential clinical scenarios to represent the range of circumstances that might present to a clinician. The panellists then rated each scenario on a scale of 1 to 9, with 1 meaning very inappropriate and 9 very appropriate (round 1). Appropriate is defined to mean that the likely benefits exceed the likely risks by a sufficient margin that the procedure is worth doing. The experts then met in a face to face meeting in March 2000. At this meeting, the scenarios were discussed and modified as needed and finally reassessed on the same nine point scale (round 2).

Even though the overwhelming majority of candidates for tubes present with acute otitis media or otitis media with effusion, the inclusion of detailed clinical factors such as age, duration and laterality of effusion, frequency of acute otitis media, extent of hearing loss, otoscopic findings, speech delay, and presence of significant disruption of family life resulted in 2268 permutations, each of which was rated as described above. The results of the second round are reported as the panel’s findings, with the median score representing the overall finding. We interpreted scores of 1, 2, and 3 as inappropriate; 7, 8, and 9 as appropriate; and 4, 5, and 6 as of equivocal or uncertain appropriateness. For this study, we considered three or more panellists rating a scenario 7-9 and three or more rating it 1-3 to represent significant disagreement and interpreted tubes for children who presented with these scenarios as of uncertain appropriateness.

The 1994 guideline was developed independently of this study by the three clinical societies and was published as a clinical practice guideline by the US Agency for Health Care Policy and Research.^25^ The 1994 guideline was limited to “healthy” children from their first birthday until before they turn 4 and suggests that insertion of a tympanostomy tube is optional after three months of persistent effusion with bilateral hearing loss and is recommended after four to six months of bilateral effusion.

### Analysis

We mapped each child to the detailed clinical scenario rated by the panel that was consistent with the details of the clinical history. We mapped children with both acute otitis media and otitis media with effusion to two clinical scenarios, one for which acute otitis media predominates and one for which otitis media with effusion predominates. The scenario with the higher (more appropriate) rating yielded the appropriateness rating for that particular child’s tympanostomy. We considered surgeries identified as appropriate or uncertain to be concordant with the explicit criteria; others were discordant.

We also compared practice with the 1994 academy guideline, which was the guideline in force at the time of our data collection. As no national guidelines on the surgical management of acute otitis media exist, we limited our analysis to children with otitis media with effusion. We considered surgeries that were either recommended or optional to be concordant with the academy guideline.

We also examined how alternatives to our expert panel’s judgment would affect the appropriateness ratings for the treatment of children with acute otitis media. Finally, we looked at the relation to appropriateness of the presence of conditions that would place a child at risk for poor outcomes, a history of tympanostomy tube surgery, and other procedures done at the time of tympanostomy tube surgery.^5^

We used χ^2^ tests to examine differences in appropriateness ratings between hospitals and subpopulations of children. Statistical analyses used Stata Statistical Software version 9.2.

## Results

### Patients’ characteristics

All children had a primary care provider, 99% of whom were paediatricians. Their mean age was 3.8 years, and 61% were white. Nearly three quarters had private insurance, and less than 5% were uninsured. More than a quarter (26.5%) of children had previously received tubes, and 21.7% had another surgery done concurrently with the insertion of a tympanostomy tube; 17.3% of children had a clinical condition that could be considered to place them “at risk” of poor developmental outcomes (table 1). Children with acute otitis media on average had about three infections in the six months before tympanostomy. Children with otitis media with effusion had less than 30 consecutive days of bilateral effusion before tympanostomy (table 2). Only 25% of children with otitis media with effusion had bilateral effusions of more than 49 days’ duration at surgery. More than a quarter of children who had surgery had normal audiograms.^27^

**Table 1 tbl1:** Sociodemographic and clinical characteristics. Values are numbers (percentages) unless stated otherwise

Characteristics	Value (n=682)
Mean age (years)	3.8
Median (range) age (years)	3.3 (0.5-13.6)
Female	292 (42.8)
Race:	
White	416 (61.0)
Black	44 (6.4)
Hispanic	82 (12.0)
Other (Asian, Pacific Islander )	39 (5.7)
Not reported	101 (14.8)
Insurance:	
Medicaid	142 (20.8)
Private	511 (74.1)
Other*	29 (4.2)
Previous tubes	181 (26.5)
Any procedure at time of tube insertion†	148 (21.7)
At risk conditions‡	118 (17.3)
Any abnormal audiogram during entire year	495 (72.5)
Speech delay	195 (28.5)
Marked otoscopic findings§	23 (3.3)
Severe disruption of family life	15 (2.2)
History of unilateral or bilateral otitis media	254 (37.3)

*Self pay and no data.

†Includes otoscopy, external ear incision, tympanoplasty; head and neck procedures include laryngoscopy, tracheotomy, tooth extraction, ophthalmoscopy, and anaesthetised eye examination among other procedures; below neck procedures include venous catheter placement, hernia repair, circumcision, and orchiopexy among many other procedures.

‡Includes children with hearing loss independent of otitis media with effusion; language or speech disorder; autism and other developmental symptoms; and Down’s syndrome or other craniofacial syndromes that include cognitive, speech, or language delay, visual impairment, cleft palate, and developmental delay.

§Presence of any of severe tympanic membrane retraction, atelectasis or myringostapediopexy.

**Table 2 tbl2:** Clinical characteristics of cohort (year before tympanostomy)

Characteristics	Mean (SE)	Median (interquartile range)
Episodes of infection*		
6 months before tympanostomy	3.1 (0.1)	3 (2-4)
1 year before tympanostomy	4.6 (0.1)	4 (3-6)
**Length of effusion†**		
Consecutive days of effusion‡:		
Bilateral	30.7 (1.7)	16 (0-49)
Unilateral (left)	39.1 (2.0)	26 (2-63.5)
Unilateral (right)	39.8 (2.0)	28 (3-63.5)
Cumulative days of effusion§:		
Bilateral	77 (2.9)	66 (30-109.5)
Unilateral (left)	91.7 (3.2)	78.5 (40.5-131.5)
Unilateral (right)	96.8 (3.1)	88 (46.5-138)

*Children with acute otitis media (n=230).

†Children with otitis media with effusion (n=452).

‡Refers to effusion directly preceding surgery.

§Cumulative effusion over one year

### Explicit criteria

Tables 3 and 4 show a sample of the 2268 scenarios. Of 2268 potential clinical scenarios, we saw 220 (9.7%) in clinical practice. Of those 220, our expert panel disagreed on four (1.7%) scenarios, which were seen in five (0.7%) children.

**Table 3 tbl3:** Appropriateness of tympanostomy tube placement* in sample of indications rated by expert panel (otitis media with effusion)

Indications	Duration of current middle ear effusion†				
	<60 days	60-90 days	91-120 days	121-180 days	>180 days
No hearing test and:					
1. No antibiotics for the effusion	Inappropriate	Inappropriate	Inappropriate	Inappropriate	Uncertain
2. One or more courses of antibiotics	Inappropriate	Inappropriate	Inappropriate	Uncertain	Uncertain
Normal hearing test and:					
3. No antibiotics for the effusion	Inappropriate	Inappropriate	Inappropriate	Uncertain	Uncertain
4. One or more courses of antibiotics	Inappropriate	Inappropriate	Inappropriate	Uncertain	Uncertain
Unilateral abnormal hearing test and:					
5. No antibiotics for the effusion	Inappropriate	Inappropriate	Uncertain	Uncertain	Appropriate
6. One or more courses of antibiotics	Inappropriate	Inappropriate	Uncertain	Uncertain	Appropriate
Bilateral abnormal hearing test (mild) and:					
7. No antibiotics for the effusion	Inappropriate	Inappropriate	Uncertain	Appropriate	Appropriate
8. One or more courses of antibiotics	Inappropriate	Uncertain	Appropriate	Appropriate	Appropriate
Bilateral abnormal hearing test (moderate to severe) and:					
9. No antibiotics for the effusion	Inappropriate	Inappropriate	Appropriate	Appropriate	Appropriate
10. One or more courses of antibiotics	Inappropriate	Uncertain	Appropriate	Appropriate	Appropriate

The table shows a sample of 2240 clinical scenarios developed and rated for children with otitis media with effusion. Other clinical scenarios for otitis media with effusion incorporated variables such as duration of unilateral effusion, age of patient, presence of speech delay, otoscopic findings, history of otitis media, and impact of otitis media on family. Each numbered row represents five possible clinical scenarios.

*In patients with bilateral persistent otitis media with effusion, no history of middle ear disease before the current episode, age less than 3 years, no speech delay, absence of marked otoscopic findings, and absence of severe disruption of family life.

†Refers to consecutive days of effusion directly preceding surgery.

**Table 4 tbl4:** Appropriateness of tympanostomy tube placement in patients with recurrent acute otitis media

Indications	Frequency of occurrence*	
	Low	High
A. Absence of severe disruption of family life and:		
A1. No antibiotic prophylaxis	Inappropriate	Uncertain
A2. Short term antibiotic prophylaxis and no otitis media on prophylaxis	Inappropriate	Uncertain
A3. Short term antibiotic prophylaxis and otitis media within one month of discontinuing prophylaxis	Inappropriate	Appropriate
A4. Short term antibiotic prophylaxis and otitis media on prophylaxis	Uncertain	Appropriate
A5. Long term antibiotic prophylaxis and no otitis media on prophylaxis	Inappropriate	Inappropriate
A6. Long term antibiotic prophylaxis and otitis media within one month of discontinuing prophylaxis	Inappropriate	Appropriate
A7. Long term antibiotic prophylaxis and otitis media on prophylaxis	Uncertain	Appropriate
B. Severe disruption of family life and:		
B1. No antibiotic prophylaxis	Inappropriate	Appropriate
B2. Short term antibiotic prophylaxis and no otitis media on prophylaxis	Inappropriate	Uncertain
B3. Short term antibiotic prophylaxis and otitis media within one month of discontinuing prophylaxis	Uncertain	Appropriate
B4. Short term antibiotic prophylaxis and otitis media on prophylaxis	Uncertain	Appropriate
B5. Long term antibiotic prophylaxis and no otitis media on prophylaxis	Inappropriate	Inappropriate
B6. Long term antibiotic prophylaxis and otitis media within one month of discontinuing prophylaxis	Inappropriate	Appropriate
B7. Long term antibiotic prophylaxis and otitis media on prophylaxis	Uncertain	Appropriate

*Acute otitis media considered to be of high frequency if at least four episodes of acute otitis media had occurred in the six months preceding surgery, or six or more episodes in the year before surgery with at least two episodes in the six months preceding surgery; otherwise frequency was considered to be low.

### Analysis based on explicit criteria

The explicit criteria classified 7.0% (48 cases) of actual surgeries as appropriate, 23.3% (159 cases) as of uncertain appropriateness, and 69.7% (475 cases) as inappropriate (table 5). Cases that were classified as appropriate, uncertain, and inappropriate had on average 80, 38, and 18 days of effusion. Counting surgeries classified as appropriate or uncertain as concordant, 30.3% were concordant with the explicit criteria. We found no statistically significant differences in appropriateness between hospitals.

**Table 5 tbl5:** Appropriateness ratings based on explicit criteria and academy guidelines. Values are numbers (percentages)

	Concordant			Not concordant
	Appropriate	Uncertain		Inappropriate
**Ratings according to explicit criteria**				
All children (n=682)	48 (7.0)	159 (23.3)		475 (69.7)
Acute otitis media (n=230)	9 (3.9)	110 (47.8)		111 (48.3)
Otitis media with effusion (n=452)	39 (8.6)	49 (10.8)		364 (80.6)
All children: criteria modified to eliminate preference for antibiotic prophylaxis* (n=682)	150 (21.9)	57 (8.3)		475 (69.7)
Excluding cases with potentially extenuating circumstances† (n=341)	31 (9.1)	104 (30.5)		206 (60.4)
**Academy guidelines‡**				
1994 Academy guidelines (n=172)	13 (7.5)			159 (92.5)
1994 Academy guidelines (age >1 year) (n=533)	30 (5.6)			503 (94.4)

*The expert panel considered indication for surgery for recurrent acute otitis media to be of uncertain appropriateness no antibiotic prophylaxis was used to suppress reoccurrence; this analysis elevates the ratings for surgeries that failed to meet this aspect of the criteria from uncertain to appropriate (a potentially alternative view); no official guidelines on the surgical treatment of acute otitis media exist.

†Such as history of previous tubes, other surgery/procedure at time of tube insertion and “at risk conditions”; the expert panel considered only the nature and magnitude of ear disease in the decision to insert tubes and not consider these extenuating circumstances.

‡The 1994 Academy guideline covers the management of otitis media with effusion, not recurrent acute otitis media.

*Cases classified with otitis media with effusion*—Overall, 80% of the cases with otitis media with effusion were not concordant with the explicit criteria developed by the panel (table 5). Among children with effusion as the reason for surgery, 76% of the inappropriate cases were inappropriate primarily owing to the short duration of the effusion immediately preceding surgery.

*Cases classified with acute otitis media*—Overall, 48% of the cases were not concordant with the explicit criteria developed for acute otitis media (table 5). Low frequency of infection was the most common reason why the cases were not concordant with the explicit criteria. The expert panel believed that the benefit of delaying surgery until after a failure of antibiotic prophylaxis for recurrent acute otitis media outweighed concerns about the development of antimicrobial resistance. Evolving views on the use of antibiotics suggested that we should also present our analysis as if the expert panel had reversed its judgment,^29^ and we reanalysed the data considering frequently recurrent acute otitis media without a trial of prophylaxis to be an appropriate indication for insertion of a tympanostomy tube rather than an uncertain one. This increased the overall proportion of appropriate cases from 7% to 22.1%; the proportion concordant remained unchanged (table 5).

### Analysis based on academy guidelines

The 1994 guideline was concerned with healthy children aged 1-3 years with otitis media with effusion: 172 children in our sample met these criteria. Among these 172 cases, 7.5% of tympanostomy tube insertions were concordant with the guideline and 92.5% were not. If we expanded the sample to include all 533 healthy children older than 1 year, then 5.6% of tympanostomies were concordant with the guideline (table 5). Again, the main reason for discordance with the guideline was short duration of effusion.

### Additional analyses

In an additional analysis, we excluded all children with potentially extenuating circumstances, such as a history of previous tube insertion, another surgery/procedure at the time of tube insertion (for which the tubes do not carry a marginal risk of anaesthesia), and the presence of various conditions that would place a child at risk of a poor developmental outcomes from the sample. We found that the explicit criteria would consider 9.1% of the surgeries to be appropriate, 30.5% to be of uncertain appropriateness, and 60.4% to be inappropriate. Thus even in a liberal review, more than 60% of cases were not concordant with the panel’s findings (table 5). Not surprisingly, concordance with the criteria also was lower for children in each of these three subpopulations than for the population as a whole (table 6).

**Table 6 tbl6:** Variability of concordance with explicit criteria

Subpopulations of children	Concordant (%)	P value
All children (n=682)	207 (30.3)	
At risk		<0.0001
Yes (n=118)	18 (15.2)	
No (n=564)	189 (33.5)	
Concomitant procedure:		<0.05
Yes (n=148)	34 (22.9)	
No (n=534)	173 (32.4)	
History of tympanostomy tubes:		<0.001
Yes (n=181)	37 (20.4)	
No (n= 501)	170 (33.9)	

*Includes children with hearing loss independent of otitis media with effusion; language or speech disorder; autism and other developmental symptoms; Down’s syndrome or other craniofacial syndromes that include cognitive, speech, or language delay, visual impairment, cleft palate, and developmental delay.

## Discussion

Using data collected from physicians, we found that tympanostomy tubes in the United States are often used in a manner inconsistent with expert recommendations. More than a decade later, this supports the 1994 report of inappropriate use of tympanostomy tubes.^4^ Although our findings are based on a local sample, the finding that practice is discordant from recommendations seems to be robust to time, to method of data collection, and to choice of expert standard.

### Implications of findings

The finding that 69% of cases deviated from the practices specifically developed for this study—or that more than 92% of surgeries would have been “not recommended” according to the guideline in force at the time of the surgery—suggests considerable overuse of this procedure. Our data suggest that children often receive tympanostomy tubes for effusions of short duration in a manner that is inconsistent with expert judgment. The experts who developed the academy guideline and the explicit criteria explicitly sought to balance the risks and benefits of the procedure. Historically, the major benefit of tympanostomy tubes discussed in the literature pertains to speech and language development. Recent research provides strong evidence that delay in the insertion of tympanostomy tubes is not associated with worse behavioural or developmental outcomes.^30^ ^31^ However, these findings do not imply that tubes should be avoided or that there are not health systems for which tubes may be underused.

What if the major benefit of tubes is not in terms of promoting enhanced development but in terms of improving functional status or quality of life? Limited evidence shows that tubes improve disease specific quality of life.^32^ ^33^ ^34^ If tubes do make children feel better or otherwise improve the quality of their lives in the short term, then the emphasis on long term outcomes and development that has predominated in the guideline may not be sufficient.

Our data also show that otolaryngologists treat children differently if they have one or more of three specific circumstances—a history of previously having had tympanostomy tubes, the scheduling of a concomitant surgery, and the presence of one or more of the conditions we identified as putting a child “at risk.” In these three situations, children receive tympanostomy tubes with less current disease. About half of our sample had one or more of these circumstances present. The extent to which these circumstances should be extenuating is questionable.

The assessment of the use of tubes in children with recurrent acute otitis media is not covered by clinical society guidelines, and the research evidence of efficacy is sparse. The explicit criteria developed by the panel of experts we convened generally indicate that tympanostomy tubes should be reserved for children with at least six episodes of recurrent acute otitis media in 12 months, who had had at least one infection that broke through antibiotic prophylaxis. The use of prophylactic antibiotics has fallen out of favour because of concerns about antibiotic resistance rather than controversy about its effectiveness. When we reanalysed the data, dropping the panel’s requirement for a failure of antibiotic prophylaxis, a substantial majority still failed to meet the standard for sufficient frequency and were still considered inappropriate.

### Strengths and limitations

In this study, we focused only on children who had received tubes; we did not consider the possible underuse of tympanostomy in some populations. We restricted our analyses to those children for whom we had complete data (a strength), but our timing during implementation of a US federal privacy rule restricted our access to charts for about one third of children (a weakness). However, children with complete data were similar to those without complete data in terms of sociodemographic characteristics available in the hospital medical record.^27^ The finding that the insertion of tympanostomy tubes is often inappropriate is robust to the extent of missing data in our study. Even considering the most generous assumption that insertion of a tympanostomy tube was appropriate for every child with data missing, more than 40% of cases would still be considered inappropriate. Therefore, the missing data would not change the overall conclusions.

To make assessments about the course of otitis media we needed to translate the intermittent clinical assessments available from the charts into the continuous variables (days of effusion) that we used in our analysis. The need to impute findings is an unavoidable limitation; however, as we described in the methods, we made generous clinical assumptions that would favour a longer duration of effusion and concordance with the explicit criteria and the academy guideline. The data came from medical record notes with all the limitations therein. We recognise that variables such as “severe disruption of family life” that rely on a physician’s notation of the impact of the disease on the family may not be regularly documented in the medical record. However, changes in the frequency with which this variable was observed would probably not alter the conclusions; the duration of effusion was too short in the vast majority of the cases.

A significant strength of this research is that we used expert recommendations from two sources to examine the concordance between practice and guidelines. To our knowledge this is the only study on appropriateness of tympanostomy tube insertion that has been done in the past decade.^4^ Although this study was local in nature, it focused on the most populous metropolitan area in the United States and is probably representative of other urban areas in the country. In addition, this study is the first to use data independently collected from community physicians’ medical records^4^; the cost of doing a national study of this scope would have been prohibitive.

### Conclusions

Regardless of whether current practice represents a substantial overuse of surgery or the guidelines are overly restrictive, the persistent discrepancy between guidelines and clinical practice cannot be good either for children or for those interested in improving their health. Substantial overuse would expose children to risk and consume resources that could be better applied to otherwise improving the health of children. Erroneous guidelines could lead clinicians, policy makers, and researchers to ill advised interventions and undermine the value of guidelines in general. Given the ubiquity of this disease and its surgical treatment, resolution of these issues should represent an urgent priority. The UK experience may prove a useful resource for policy makers in the United States as they take on these challenges.

**What is already known on this topic**

Tympanostomy tubes are used commonly in the United States to treat otitis media, whereas in the UK this practice has been reduced
A 1994 study which suggested that inappropriate use was common in the US was controversial because of its data sources and the criteria used

**What this study adds**

Most insertions of tympanostomy tubes in the New York metropolitan area were for inappropriate reasons according to two different standards
These findings suggest a serious discrepancy between the clinical care of children and the recommendations that experts suggest should be the standard of care in the US

## Notes

Expert panel: Ellen M Friedman, chief, Pediatric Otolaryngology, Texas Children’s Hospital, Houston, TX; G Scott Giebink, professor of pediatrics and otolaryngology, University of Minnesota Medical School, Minneapolis, MN; Gregory Hayden, professor of pediatrics, University of Virginia Health System, Charlottesville, VA; John Hickner, professor and vice chair, Department of Family Medicine, University of Chicago Pritzker School of Medicine; Margaret A Kenna, professor of otology and laryngology, Harvard Medical School, Boston, MA; Jack L Paradise, professor of pediatrics, University of Pittsburgh, Pittsburgh, PA; Seth H Pransky, director, Pediatric Otolaryngology, Children’s Hospital, San Diego, CA; Oliver Roddey, Eastover Pediatrics, Charlotte, NC. One panel member from the Seattle, WA, area preferred to remain anonymous.

Ethical approval: Institutional review boards of all five hospitals. This is a retrospective medical record review and no contact with patients was permitted.

This work was presented at the June 2007 and 2008 annual AcademyHealth meetings.

Provenance and peer review: Not commissioned; externally peer reviewed.

## Notes

**Cite this as: ***BMJ* 2008;337:a1607
